# CD73-dependent generation of extracellular adenosine by vascular endothelial cells modulates *de novo* lipogenesis in adipose tissue

**DOI:** 10.3389/fimmu.2023.1308456

**Published:** 2024-01-09

**Authors:** Michelle Y. Jaeckstein, Isabell Schulze, Michael Wolfgang Zajac, Markus Heine, Oliver Mann, Alexander Pfeifer, Joerg Heeren

**Affiliations:** ^1^ Department of Biochemistry and Molecular Cell Biology, University Medical Center Hamburg-Eppendorf, Hamburg, Germany; ^2^ Department of General, Visceral and Thoracic Surgery, University Medical Center Hamburg-Eppendorf, Hamburg, Germany; ^3^ Institute of Pharmacology and Toxicology, University Hospital, University of Bonn, Bonn, Germany

**Keywords:** white adipose tissue, endothelial cells, adenosine, CD73, purinergic signaling, intercellular crosstalk, *de novo* lipogenesis

## Abstract

Next to white and brown adipocytes present in white and brown adipose tissue (WAT, BAT), vascular endothelial cells, tissue-resident macrophages and other immune cells have important roles in maintaining adipose tissue homeostasis but also contribute to the etiology of obesity-associated chronic inflammatory metabolic diseases. In addition to hormonal signals such as insulin and norepinephrine, extracellular adenine nucleotides modulate lipid storage, fatty acid release and thermogenic responses in adipose tissues. The complex regulation of extracellular adenine nucleotides involves a network of ectoenzymes that convert ATP via ADP and AMP to adenosine. However, in WAT and BAT the processing of extracellular adenine nucleotides and its relevance for intercellular communications are still largely unknown. Based on our observations that in adipose tissues the adenosine-generating enzyme CD73 is mainly expressed by vascular endothelial cells, we studied glucose and lipid handling, energy expenditure and adaptive thermogenesis in mice lacking endothelial CD73 housed at different ambient temperatures. Under conditions of thermogenic activation, CD73 expressed by endothelial cells is dispensable for the expression of thermogenic genes as well as energy expenditure. Notably, thermoneutral housing leading to a state of low energy expenditure and lipid accumulation in adipose tissues resulted in enhanced glucose uptake into WAT of endothelial CD73-deficient mice. This effect was associated with elevated expression levels of *de novo* lipogenesis genes. Mechanistic studies provide evidence that extracellular adenosine is imported into adipocytes and converted to AMP by adenosine kinase. Subsequently, activation of the AMP kinase lowers the expression of *de novo* lipogenesis genes, most likely via inactivation of the transcription factor carbohydrate response element binding protein (ChREBP). In conclusion, this study demonstrates that endothelial-derived extracellular adenosine generated via the ectoenzyme CD73 is a paracrine factor shaping lipid metabolism in WAT.

## 1 Introduction

Adipose tissues critically regulate metabolic, endocrine as well as immune modulatory responses (1–4). In mammals, two distinct types of adipocytes known as white and brown are recognized to significantly contribute to energy metabolism (5). In brown adipose tissue (BAT), thermogenic brown adipocytes produce heat to regulate body temperature in cold environments, an energy-intensive process referred to as adaptive thermogenesis (6–10). Under certain catabolic conditions, beige adipocytes can develop in different white adipose tissue (WAT) depots. It is noteworthy that these thermogenic cells resemble brown adipocytes both morphologically and functionally (6, 11–13). These thermogenic adipocytes are characterized by a high mitochondrial number, high oxidative capacity and the expression of the uncoupling protein-1 (UCP1). At the inner mitochondrial membrane, this protein enables heat production by separating the proton gradient generated by the respiratory complexes from ATP production (7, 14–16). In response to cold ambient temperature, activation of the autonomous nervous system results in norepinephrine secretion at the terminal sympathetic nerve ends innervating BAT. This initiates beta-adrenergic signalling, which promotes lipolysis of triglycerides by the intracellular lipases, mitochondrial respiration and UCP1-dependent heat production (17, 18). In parallel, energy stores of thermogenic adipocytes are replenished by high disposal of fuels such as fatty acids delivered by triglyceride-rich lipoproteins and glucose. The uptake of these energy-rich substrates is regulated primarily by insulin-driven pathways and beta-adrenergic signalling (10, 19, 20).

Energy uptake and fuel oxidation by thermogenic adipocytes contributes substantially to systemic energy homeostasis in mice and humans, and clinical studies suggest that active BAT has a number of beneficial effects on metabolism in various organs including liver, muscle and heart (6, 7, 21–24). White adipocytes store large amounts of triglycerides, which can be mobilized by intracellular lipases to provide other metabolically active organs with energy. Similar to brown adipocyte activation, this hydrolytic process is activated mainly via norepinephrine-stimulated adrenergic signalling (25). Fatty acids liberated by WAT include exogenous ones originally derived from the diet and such from endogenous synthesis produced under conditions of caloric excess (26). Dietary lipids are delivered by intestinal lipoproteins known as chylomicrons. These triglyceride-rich particles are hydrolysed at the vascular endothelium of WAT by the enzyme lipoprotein lipase. Subsequently, the released free fatty acids are internalized and re-esterified to form triglycerides that are stored in lipid droplets of white adipocytes (19, 27–30). For endogenous fatty acid synthesis, dietary glucose is metabolized to acetyl-CoA and then converted to fatty acids by a series of enzymatic steps mediated by acetyl-CoA carboxylase alpha (ACC) and fatty acid synthase (FASN). This process known as *de novo* lipogenesis (DNL) is triggered by insulin-dependent glucose uptake and regulated by the transcription factors sterol regulatory element-binding protein (SREBP) and the two isoforms of the carbohydrate responsive element-binding protein (ChREBP) (31–35). Notably, ChREBP-alpha is activated by dephosphorylation upon glucose uptake and determines the expression of the constitutively active isoform ChREBP-beta (31, 32, 36).

Next to insulin and norepinephrine, paracrine factors released by various cell types present in WAT and BAT are involved in the regulation of glucose and lipid uptake, fatty acid release and energy expenditure (37, 38). In addition to peptides and lipokines, extracellular adenine nucleotides such as adenosine were suggested to shape intercellular crosstalk as well as thermogenic and metabolic function in adipose tissues (39, 40). For instance, pharmacological activation of adenosine receptor signalling promotes browning behaviour and stimulates thermogenesis in BAT and WAT, thereby combating obesity and associated metabolic disturbances (41–43). Physiologically, adenosine is produced by the stepwise hydrolysis of extracellular ATP, a process mediated by two ectonucleotidases. The ectonucleoside triphosphate diphosphohydrolase-1 (referred to as CD39) hydrolyses ATP via ADP to AMP, while the ectoenzyme CD73 facilitates the subsequent reaction from AMP to adenosine (44).

In this study, we investigate the physiological role of CD73 in BAT and WAT under conditions of thermogenic activation and thermoneutrality. In particular, we show that the expression of CD73 is mainly found in tissue-resident endothelial cells of adipose tissue depots. Based on this finding, we generated an endothelial cell-specific CD73-deficient mouse model. Under cold exposure conditions, endothelial CD73 was dispensable for thermogenic adaptation of adipose tissues. On the other hand, in mice exposed to thermoneutrality, endothelial CD73 deficiency resulted in increased glucose uptake, which was associated with increased DNL gene expression in WAT. Mechanistically, we provide evidence that extracellular adenosine is imported by adipocytes and converted to AMP by adenosine kinase. Subsequently, activated AMP kinase (AMPK) lowers ChREBP target genes, including the expression of ACC and FASN, at both mRNA and protein levels. Overall, our data highlight the importance of intercellular crosstalk between endothelial cells and adipocytes via adenosine in regulating glucose uptake and lipid storage of WAT.

## 2 Materials and methods

### 2.1 Mice

All experiments were approved by the Behörde für Gesundheit und Verbraucherschutz Hamburg (N082/2020). Mice were housed at the UKE animal facility at room temperature and a 12h light/12h dark cycle with permanent access to food and water. C57BL/6 wild type mice were bred in-house or obtained from Charles River. Prof. Juergen Schrader provided Cd73^fl/fl^ mice (University Hospital of Duesseldorf, Germany), which were crossed with Tg(Cdh5-cre/ERT2)1Rha mice (45). By this breeding, we generated Cd73^fl/fl^-Cdh5^Cre+^ and control litters (Cd73^fl/fl^-Cdh5^Cre-^). To induce the endothelial-specific Cd73-knockout, the mice were gavaged on 3 consecutive days with 100 µl corn oil containing 2 mg tamoxifen. After two weeks, the mice received another tamoxifen gavage to deplete CD73 also in newly-formed endothelial cells. Age-matched male and female mice (10-16 weeks) were used for the experiments. For cold exposure and for thermoneutral studies, mice were exposed to cold (6°C), at mild cold (22°C) or housed at thermoneutrality (30°C) for 1 week, respectively, using a temperature- and humidity-controlled cabinet (Memmert). For organ harvest and blood collection, mice were anaesthetised by ketamine (180 mg/kg) and xylazine (24 mg/kg) injection after 4 h fasting.

### 2.2 Human WAT samples

The Ethics Committee of the Hamburg Chamber of Physicians approved the study (PV4889). For the isolation of stromal vascular fractions, WAT samples from three male patients with morbid obesity (age between 36 and 45 years) during bariatric surgery were harvested at the Department of General, Visceral and Thoracic Surgery, University Medical Center Hamburg-Eppendorf. All those participating signed and returned an informed consent.

### 2.3 Isolation of adipocytes, tissue-resident macrophages and endothelial cells

Cell types were isolated from WAT and BAT as described previously (46). In brief, inguinal depots of WAT or interscapular depots of BAT of 4 mice were pooled and digested for 45 min using a collagenase D (1.5 U/ml) and dispase II (2.4 U/ml) containing buffer. After filtering through a 100 µm cell strainer, the flow through was pelleted by centrifugation for 5 min, at 600 x g. The remaining sample was resuspended (PBS containing 2 mM EDTA, 0.5% BSA, 2 mM glucose), passed through a 40 µm cell strainer and centrifuged (5 min, 600 x g). The cell pellet was resuspended and incubated for 15 min with CD11b MicroBeads (Miltenyi, 10 µl beads/10^7^ cells) for isolation of the macrophage fraction. The CD11b+-fraction was separated using magnetic LS-columns (Miltenyi). The remaining sample was incubated with CD31 MicroBeads (Miltenyi) for endothelial cell isolation. The remaining flow through fraction was considered as adipocyte pool. Cell fractions were centrifuged and the obtained pellets dissolved for RNA isolation in TRIzol^®^ reagent.

### 2.4 Gene expression analysis

RNA was isolated from whole organ samples, BAT and WAT cell fractions or primary adipocyte cell cultures using TRIzol^®^ reagent and a NucleoSpin RNAII kit (Macherey & Nagel) as described in protocol of the company. RNA concentration was determined using NanoDrop^®^ spectrophotometer. Then, 400 ng of RNA was transcribed into cDNA (High-Capacity cDNA Reverse Transcription Kit, Applied Biosystems) following the manufacturer’s instructions. Quantitative real-time RT-PCR was performed on a QuantStudio 5 Real- Time-PCR System (ThermoFischer Scientific) using TaqMan^®^ Assay-on-Demand primer sets. Taqman^®^ assays used in this study: *h36B4* (Hs99999902_m1), *hACACA* (Hs01046047_m1), *mAcaca* (Mm01304285_m1), *hCD36* (Hs00169627_m1), *mCd36* (Mm00432403_m1), *hCHREBP* (Hs00975710_g1), *mChrebp* (Mm00498811_m1), hCHREBP_BETA (AIT9559), *mChrebp_beta* (AIVI4CH), *mDio2* (Mm00515664_m1), *hFASN* (Hs00188012_m1), *mFasn* (Mm00662319_m1), *hGLUT4* (Hs00168966_m1), *mGlut4* (Mm01245502_m1), *hLPL* (Hs00173425_m1), *mLpl* (Mm00434764_m1), *mNt5e* (Mm00501910_m1), *hSREBF1* (Hs01088691_m1), *mSrebf1* (Mm00550338_m1), *hSREBP1C* (AI70MDO), *mSrebp1c* (AI89KJW), *hSREBP2* (Hs01081778_m1), *mSrebf2* (Mm01306292_m1), *mTbp* (Mm00446973_m1), *mUcp1* (Mm00494069_m1). Relative mRNA-expression was normalized to *mTbp* (TATA-box binding protein) or *h36B4* (hRPLP0= ribosomal protein lateral stalk subunit P0) as housekeeper and calculated via ΔΔCT method.

### 2.5 Glucose and lipid uptake studies

For oral glucose fat tolerance test (OGFT), mice received orally a lipid-glucose-emulsion containing glucose (2 mg/g body weight) and triglycerides (3.7 mg/g body weight), containing ^14^C-deoxyglucose (1.7 kBq/g body weight) and ^3^H-triolein (1.7 kBq/g body weight) as radiolabelled tracers after 2 hours of fasting. At time points indicated in the figures, glucose concentrations were determined in blood taken from tail vein using commercially available AccuCheck Aviva glucose sticks (Roche). Then, mice were anaesthetized and tissues were collected after transcardial perfusion with phosphate buffer saline containing 10 U/ml heparin. After dissolving organs using Solvable™, radioactivities were determined using liquid scintillation counting (Tricarb, Perkin Elmer).

### 2.6 Indirect calorimetry

Indirect calorimetry was performed in metabolic cages (PhenoMaster, TSE systems). For data acquisition, mice were exposed to various temperatures to monitor oxygen consumption and carbon dioxide production. Based on respiratory data, energy expenditure was calculated as kcal per hour.

### 2.7 Plasma parameters

Plasma lipid levels (cholesterol, triacylglycerides) were measured by enzymatic-colorimetric assays (Roche) following the instructions of the manufacturer.

### 2.8 Cell culture

For primary brown and white adipocyte cell culture, stromal-vascular fractions (SVF) from BAT and WAT of wild type mice (C57BL/6J, male, age 4-6 weeks) were isolated as described previously (47). To obtain mature adipocyte, cells from SVF were cultured in DMEM/high glucose GlutaMAX (Gibco) supplemented with 10% neonatal calf serum, 1% penicillin and streptomycin, 1% antibiotic-antimycotic, 2.4 nM of insulin, and either 1 µM (brown adipocytes) or 100 nM (white adipocytes) of rosiglitazone (Cayman Chemicals).

For primary human white adipocyte cell culture, stromal-vascular fractions of subcutaneous WAT were isolated (as described for murine cells above) and expanded in DMEM/high glucose GlutaMAX (Gibco) containing 20% FCS (Gibco). Cells were differentiated to mature adipocytes in DMEM/high glucose GlutaMAX supplemented with 5% FCS, 1% penicillin and streptomycin, 0,1 µM dexamethasone (Sigma), 450 µM IBMX (Sigma), 2 µM insulin (Sigma), 1 µM rosiglitazone (Cayman Chemicals) and 1 µM U0126 (Sigma).

During adipogenic differentiation, cells were incubated with/without adenosine (10 µM) and/or the adenosine deaminase (ADA) inhibitor erythro-9-(2-hydroxy-3-nonyl)adenine (EHNA, 10µM) and/or adenosine kinase (ADK) inhibitor 5-(3-Bromophenyl)-7-[6-(4-morpholinyl)-3-pyrido[2,3-d]byrimidin-4-amine hydrochloride (ABT-702, 100 nM). Media change was performed every day. Adipocytes were harvested after PBS washing in TRIzol^®^ reagent for RNA isolation or in RIPA buffer (20 mM Tris-HCl, pH 7.4; 5 mM EDTA; 50 mM NaCl, 10 mM Na-Pyrophosphate; 50 mM NaF, 1% Nonidet P40) for protein analysis.

### 2.9 Western blotting

Snap-frozen adipose tissues were lysed in RIPA buffer supplemented with protease inhibitors (Complete Mini protease inhibitor cocktail; Roche), 0.1% SDS and phosphatase inhibitors (1% phosphatase inhibitor A + B; bimake) using the TissueLyser (Qiagen). Protein concentrations were determined using the BCA method. Protein lysates were separated by SDS-PAGE (10% acrylamide gels) and for Western blotting, transferred to nitrocellulose membranes. After blocking in 5%-milk in TBS-T (20 mM Tris, 150 mM NaCl, 0.1% (v/v) Tween 20) or ROTI^®^Block (ROTH; phosphorylated proteins) for 1 h, the membranes were incubated with the indicated primary antibodies over night at 4°C (diluted in 5% BSA in TBS-T). Secondary HRP-conjugated antibodies were diluted 1:5000 and incubated for 1 h. Following primary antibodies were used: rabbit-anti-ACC (1:1000; Cell Signaling #3662; RRID : AB_2219400), rabbit-anti-pACC (1:500; Cell Signaling #3661; RRID : AB_330337), rabbit-anti-AMPKα (1:1000; Cell Signaling #2532; RRID : AB_330331), rabbit-anti-pAMPKα (1:1000; Cell Signaling #2531; RRID : AB_330330), rabbit-anti-ChREBP (1:1000; Novus #NB400-135; RRID : AB_10002435), rabbit-anti-gTubulin (1:2000; Abcam #ab179503; RRID: N/A), mouse-anti-FASN (1:1000; BD Bioscience #610962; RRID : AB_398275). Following secondary antibodies were used: Peroxidase-conjugated AffiniPure goat-anti-rabbit IgG (H+L) (1:5000; Jackson ImmunoResearch #111-035-144; RRID : AB_2307391), Peroxidase-conjugated AffiniPure goat-anti-mouse IgG (H+L) (1:5000; Jackson ImmunoResearch #115-035-146; RRID : AB_10015289). The blots were developed with enhanced chemiluminescence using Amersham Imager 600 (GE Healthcare). Signal quantification was performed using ImageStudio Lite (Licor; RRID : SCR_013715).

### 2.10 Quantification and statistics

Data are expressed as mean ± S.E.M. Two groups were compared by unpaired, two-tailed Student’s t test, more than two groups were compared by ANOVA. The statistical parameters such as p values and numbers of replicates are presented in the figure legends and **Supplementary Table 1**. Statistical analysis and presentation of data were conducted using Microsoft Excel 2016 (RRID : SCR_016137) and GraphPadPrism 9.0 (RRID : SCR_002798).

## 3 Results

### 3.1 Adipose tissue CD73 is predominantly expressed by microvascular endothelial cells

**Figure 1:**
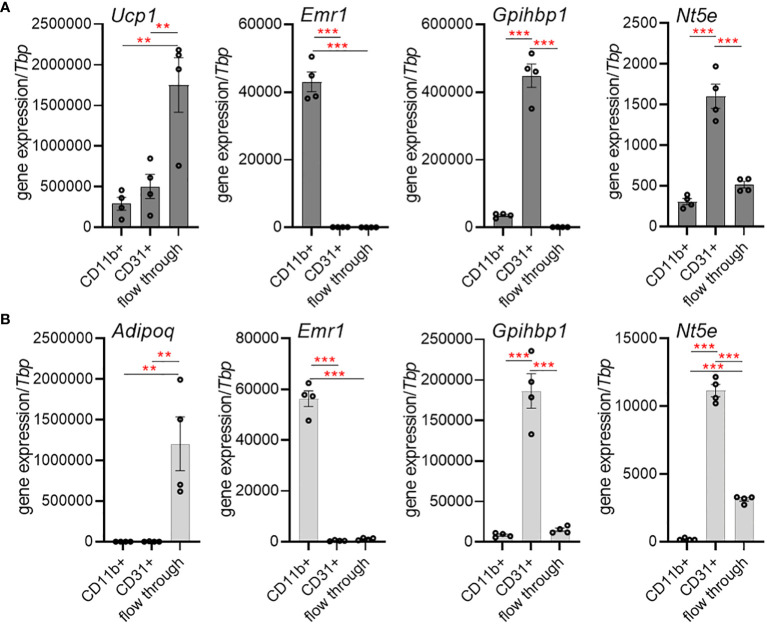
Cell-type-specific expression of CD73 (*Nt5e*) in murine brown (BAT) and white adipose tissue (WAT). **(A, B)** Gene expression of the adipocyte marker adiponectin (*Adipoq*) or the thermogenic marker uncoupling protein 1 (*Ucp1*), macrophage marker EGF-like module-containing mucin-like hormone receptor-like 1 (*Emr1*) and the endothelial marker glycosylphosphatidylinositol anchored high density lipoprotein binding protein 1 (*Gpihbp1*) in CD11b+ myeloid cells, CD31+ endothelial cells and adipocyte-enriched flow-through fraction isolated from BAT and WAT of wildtype mice using magnetic activated cell sorting (MACS). **(A)** Gene expression in BAT (*n*=4). **(B)** Gene expression in WAT (*n*=4). Data are presented as mean values ± SEM. **p < 0.01, ***p < 0.001 by ANOVA. The results indicate successful enrichment of specific cell types and higher expression of *Nt5e* in endothelial cells compared to myeloid cells and adipocytes in BAT and WAT.

As CD73 (encoded by *Nt5e*) generates extracellular adenosine, we determined the expression of *Nt5e* in murine BAT (**Figure 1A**) and WAT (**Figure 1B**) in a cell-type specific manner. For this approach, we performed magnetic-activated cell-sorting (MACS^®^) to separate adipocytes, tissue-resident macrophages and endothelial cells. The purity of the isolated cell fractions was confirmed by the expression of specific genes characteristic for brown (*Ucp1*) and white (*Adipoq*) adipocytes, macrophages (*Emr1*) and endothelial cells (*Gpihbp1*). Notably, *Nt5e* expression was predominantly detected in the endothelial cell fractions of BAT and WAT, respectively. These data suggest that primarily vascular endothelial cells of adipose tissues are equipped to generate extracellular adenosine.

### 3.2 Endothelial cell-specific CD73 deletion has minor effects on energy metabolism in cold-stressed mice

**Figure 2:**
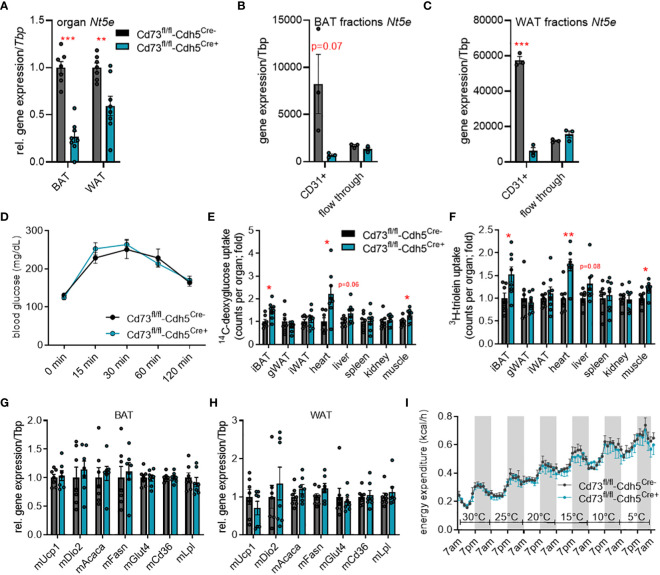
Endothelial cell-specific CD73 deletion has minor effects on energy metabolism in cold-stressed male mice. **(A-H)** Endothelial cell-specific CD73 knockout mice (CD73^fl/fl^-Cdh5^Cre+^) and controls (CD73^fl/fl^-Cdh5^Cre-^) were housed at 22°C. Successful CD73 knockdown in endothelial cells was detected by **(A)** gene expression analysis of *Nt5e* in whole brown (BAT) and white adipose tissue (WAT) (*n*=7) as well as **(B, C)** gene expression of *Nt5e* in CD31+ endothelial cells and adipocyte-rich flow-through fraction isolated from **(B)** BAT (*n*=3) and **(C)** WAT (*n*=3). **(D)** Blood glucose levels during oral glucose fat tolerance test (OGFT) (*n*=7-8). **(E, F)** Uptake of **(E)** ^14^C-deoxyglucose (DOG), and **(F)** ^3^H-triolein per total organ (*n*=7-8). **(G)** BAT and **(H)** WAT gene expression (*n*=7). **(I)** Energy expenditure in the CD73^fl/fl^-Cdh5^Cre+^ and CD73^fl/fl^-Cdh5^Cre-^ mice housed at indicated environmental temperatures in metabolic chambers (*n*=5-6). Data are presented as mean values ± SEM. *p < 0.05, **p < 0.01, ***p < 0.001 by Student’s t test. See also **Figure S1**. Overall, higher uptake of glucose and lipid tracers into BAT, heart and muscle of mice lacking endothelial CD73 expression were detected, whereas gene expression, glucose tolerance and energy expenditure were unaltered.

To study the role of vascular-derived adenosine in adipose tissue function under different environmental conditions, we generated endothelial-specific CD73-knockout mice (*CD73* ^fl/fl^-Cdh5^Cre+^) and compared them to control littermates (*CD73* ^fl/fl^-Cdh5^Cre-^). In BAT and WAT of these tamoxifen-inducible *CD73* ^fl/fl^-Cdh5^Cre+^ mice, *Nt5e* gene expression was reduced compared to controls on whole tissue level (**Figure 2A**). Successful cell type-specific deletion was confirmed in MACS^®^-isolated CD31-positive endothelial cells of BAT (**Figure 2B**) and inguinal WAT (**Figure 2C**) from *CD73* ^fl/fl^-Cdh5^Cre+^ mice. In the flow-through fractions representing adipocytes, CD73 expression was very low and unaltered between genotypes.

Previously, stimulation of the adenosine receptors, A2A and A2B, has been shown to induce thermogenesis in BAT and to stimulate browning in WAT suggesting this pathway as novel target for anti-obesity therapies (41–43). To study the potential relevance of CD73-generated adenosine in the context of thermogenic activation, we performed metabolic studies in *CD73* ^fl/fl^-Cdh5^Cre+^ and control mice housed at room temperature (22°C; mild cold for mice) as well as cold (6°C) conditions. First, to assess a potential effect on systemic glucose metabolism, we performed an oral glucose fat tolerance test (OGFT). In *CD73* ^fl/fl^-Cdh5^Cre+^ and *CD73* ^fl/fl^-Cdh5^Cre-^ mice housed at room temperature, we detected a comparable blood glucose clearance after oral gavage (**Figure 2D**). Despite similar glucose tolerance, uptake of glucose and lipid tracers into highly oxidative tissues such as BAT, heart and muscle was higher in *CD73* ^fl/fl^-Cdh5^Cre+^ mice compared to the controls (**Figures 2E, F**). However, higher fuel disposal did not affect body and organ weights as well as plasma triglyceride and cholesterol levels compared to the control mice (**Figures S1A–D**). In line, gene expression of thermogenic (*Ucp1: uncoupling protein 1, Dio2: diodinase 2*), glucose uptake (*Glut4: glucose transporter 4*) and lipid handling marker genes (*Acaca: acetyl CoA carboxylase, Fasn: fatty acid synthase, Cd36, Lpl: lipoprotein lipase*) was unaffected in BAT (**Figure 2G**) and WAT (**Figure 2H**).

To maximally challenge the thermogenic response, *CD73* ^fl/fl^-Cdh5^Cre+^ and *CD73* ^fl/fl^-Cdh5^Cre-^ mice were acclimatized to 6°C for one week. Similar to mice housed at room temperature, body and organ weights, plasma lipid levels, glucose tolerance, as well as expression of thermogenic and lipid processing genes in BAT and WAT were largely unaffected between control and endothelial cell-specific CD73-deficient mice (**Figures S1E–K**). Moreover, under sustained cold exposure conditions glucose and lipid tracer uptake into metabolically active tissues was unaltered (**Figures S1L, M**). To directly assess the relevance of endothelial CD73 in the regulation of energy expenditure during different temperature conditions, *CD73* ^fl/fl^-Cdh5^Cre+^ and control mice were housed in a temperature-controlled indirect calorimetry system. In this setup, the mice were housed at thermoneutrality (30°C) and then exposed to decreasing ambient temperatures (5°C per day) until reaching 5°C. With this approach to study systemic energy metabolism under conditions of thermogenic activation, lack in endothelial CD73 did not affect whole body energy expenditure compared to controls (**Figure 2I**). Taken together, these data indicate that endothelial CD73 is dispensable for functional thermogenic adaptation to cold conditions.

### 3.3 CD73 regulates *de novo* lipogenesis genes in WAT of thermoneutral-housed mice

**Figure 3:**
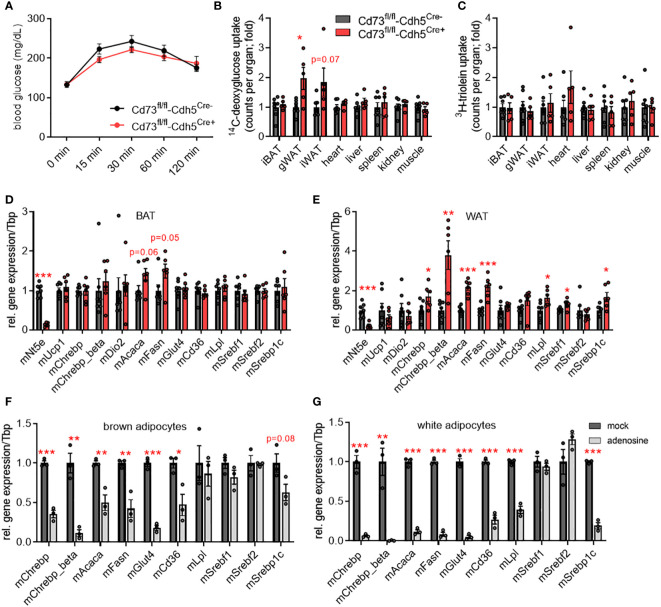
CD73 regulates *de novo* lipogenesis genes in white adipose tissue (WAT) of thermoneutral-housed mice. **(A-E)** Male endothelial cell-specific CD73 knockout mice (CD73^fl/fl^-Cdh5^Cre+^) and controls (CD73^fl/fl^-Cdh5^Cre-^) were housed at thermoneutrality (30°C). **(A)** Blood glucose levels during oral glucose fat tolerance test (OGFT) (*n*=6-7). **(B, C)** Uptake of **(B)** ^14^C-deoxyglucose (DOG), and **(C)** ^3^H-triolein per total organ (*n*=5-7). **(D)** BAT and **(E)** WAT gene expression (*n*=6-7). **(F, G)** Primary stromal-vascular cell (SVC)-derived brown and white adipocytes were differentiated in the absence (mock) or presence of adenosine. Gene expression of SVC differentiated to primary **(F)** brown and **(G)** white adipocytes (*n*=3). Data are presented as mean values ± SEM. *p < 0.05, **p < 0.01, ***p < 0.001 by Student’s t test. See also **Figure S3**. In thermoneutrally housed mice, deficiency of endothelial CD73 resulted in higher glucose uptake and elevated expression of *de novo* lipogenesis (DNL) genes in WAT depots. In primary adipocytes, adenosine supplementation causes lower expression of DNL genes.

To investigate the role of endothelial CD73 at human standard environmental temperature conditions, transgenic male mice were acclimated to thermoneutrality (30°C). Mice housed at this ambient temperature display a lower metabolic rate that more closely resembles the normal metabolic status of humans living at room temperature (48, 49). Body and organ weights as well as plasma lipid levels were similar (**Figures S2A–D**). Compared to control mice, *CD73^fl/fl^*-Cdh5^Cre+^ mice showed a slight, but not significant, improvement in blood glucose clearance after oral gavage (**Figure 3A**). Consistently, lack in endothelial CD73 resulted in higher glucose but not lipid tracer uptake into gonadal and inguinal WAT depots but not any other organs (**Figures 3B, C**). Under anabolic conditions of excess carbohydrate availability, glucose internalised by brown and white adipocytes can be used as a precursor for DNL (3). Lack in endothelial CD73 did not significantly alter the expression of DNL-regulating transcription factors and enzymes in BAT (**Figure 3D**). However and consistent with the higher glucose uptake exclusively in WAT (**Figure 3B**), gene expression of DNL-marker genes namely total *Chrebp*, *Chrebp-beta*, *Srebf1*, *Acaca* and *Fasn* was significantly higher in WAT of *CD73* ^fl/fl^-Cdh5^Cre+^ mice compared to controls (**Figure 3E**). Similar to the males, thermoneutrally housed female mice lacking endothelial CD73 are characterized by an improved glucose tolerance, displayed increased uptake of glucose tracers in adipose tissue depots and higher expression of DNL genes compared to controls (**Figures S2E–K**).

Thus, higher glucose uptake and upregulated DNL genes observed in WAT of endothelial cell-specific CD73 deficient mice could be a consequence of lower local adenosine levels. To study the direct effect of adenosine on DNL gene expression, primary brown and white murine adipocytes were differentiated in the absence or presence of 10 µM adenosine. In fact, supplementation of adenosine results in lower gene expression of *Chrebp* as well as its targets *Acaca* and *Fasn* in brown and white adipocytes compared to control (mock) treatment (**Figures 3F, G**). Moreover, glucose (*Glut4*) and lipid uptake mediators (*Cd36, Lpl*) displayed lower expression upon adenosine treatment. In contrast to the *in vivo* situation, the adenosine effect on gene expression was also present in primary brown adipocytes. To evaluate a potential impact of rapid adenosine degradation in this setup, the cell culture media was supplemented with adenosine with or without the addition of the adenosine deaminase (ADA) inhibitor EHNA (10 µM). Both conditions caused a comparable lower expression of DNL genes in brown and white adipocytes (**Figures S3A, B**). Altogether, these data suggest a paracrine signaling pathway, in which adenosine generated by CD73 of microvascular endothelial cells regulates energy handling and lipid metabolism of white adipocytes.

### 3.4 Adenosine regulates ChREBP-dependent lipid metabolism through adenosine kinase

**Figure 4:**
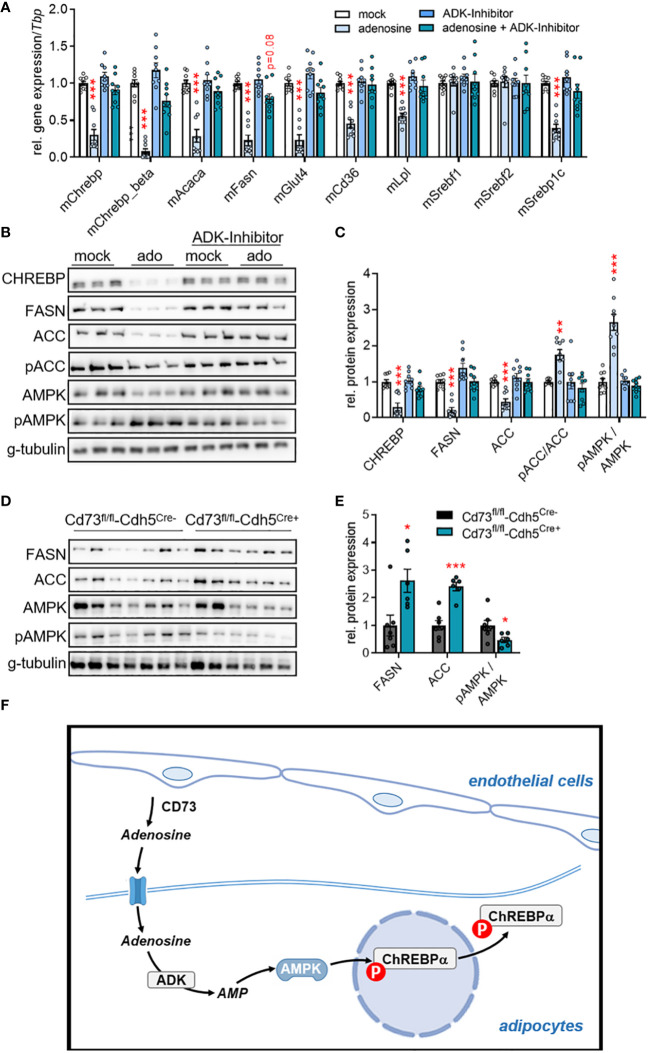
Adenosine regulates ChREBP-dependent lipid metabolism through adenosine kinase (ADK). **(A-C)** Murine primary stromal-vascular cell (SVC)-derived white adipocytes were differentiated in the absence (mock) or presence of adenosine. ADK was inhibited by ABT-702. **(A)** Gene expression (*n*=9), **(B)** representative Western blot image, and **(C)** quantification of protein expression (*n*=9). **(D, E)** Male endothelial cell-specific CD73 knockout mice (CD73^fl/fl^-Cdh5^Cre+^) and controls (CD73^fl/fl^-Cdh5^Cre-^) were housed at thermoneutrality (30°C). **(D)** Western Blot analysis of WAT samples, and **(E)** quantification of proteins shown in **(D)**. Data are presented as mean values ± SEM. *p < 0.05, **p < 0.01, ***p < 0.001 by ANOVA comparing mock versus indicated treatments or by Student’s t test comparing CD73^fl/fl^-Cdh5^Cre+^ versus CD73^fl/fl^-Cdh5^Cre-^. These data indicate that ADK inhibition rescued the lowering effect of adenosine on *de novo* lipogenesis (DNL) mediating factors on gene and protein level. **(F)** Schematic model: Vascular CD73-generated adenosine is imported by adipocytes and intracellularly converted to AMP by ADK. In turn, AMP-activated AMP kinase inhibitory phosphorylates the transcription factor carbohydrate response element binding protein (ChREBP) and thereby downregulates the expression of DNL genes.

Previous studies have shown that extracellular adenosine triggers AMP-dependent protein kinase (AMPK) activity in hepatocytes and an intestinal epithelial cell line (50, 51). Mechanistically, the authors propose that adenosine can enter the cell and is subsequently phosphorylated to AMP by the adenosine kinase (ADK). Independently of these studies, AMPK has been proposed to phosphorylate and thereby inactivate both ChREBP and ACC (52, 53). To investigate a possible link between the intracellular conversion of adenosine to AMP and ChREBP-dependent DNL, we incubated murine primary white adipocytes with adenosine in the absence or presence of 100 nM of the ADK-inhibitor ABT-702 (54). As shown in **Figures 4A–C**, adenosine supplementation resulted in lower gene and protein expression of ChREBP and DNL-mediating enzymes (ACC and FASN). Moreover, higher ratios of both pAMPK/AMPK and pACC/ACC in response to adenosine supplementation were detected (**Figures 4B, C**). Notably, the effects on gene expression, protein expression and phosphorylation were blunted by the ADK-inhibitor. These findings were validated in a human setting, where a similar modulation of CHREBP and its targets ACC and FASN by extracellular adenosine was found in primary adipocytes derived from human WAT (**Figures S4A–C**). On the other hand and consistent with lower adenosine levels, higher protein levels of ACC and FASN and a decrease in the pAMPK/AMPK ratio were detected in WAT of mice lacking endothelial CD73 (**Figures 4D, E**). Altogether, these data indicate that adenosine generated by CD73 in vascular endothelial cells shapes glucose uptake and DNL by driving an ADK-AMPK-ChREBP pathway in adipocytes and WAT depots.

## 4 Discussion

Changes in the microenvironment or dysregulated interactions between the different cell types residing in the adipose tissue are associated with pathophysiological conditions such as chronic inflammation or type 2 diabetes (55). Even though adipocytes are the main players in adipose tissue metabolism, tissue-resident endothelial cells display important functions for energy uptake, lipid storage, energy expenditure and thermogenic adaptive responses in WAT and BAT (19, 46, 56). Thus, to achieve optimal adipose tissue function under diverse metabolic challenges, it is conceivable that endothelial cells and adipocytes mutually regulate each other. However, the mechanisms of potential signalling pathways and their paracrine mediators regulating energy storage and/or combustion are still poorly defined. Previously, adenine nucleotides including adenosine and inosine were described as extracellular signalling molecules regulating function and development of adipose tissues (39–43). The generation and cellular origin of these paracrine factors remain unclear. In this study, we found the adenosine-producing ectonucleotidase CD73 to be primarily expressed by endothelial cells but not adipocytes in BAT and WAT. In metabolic studies using cell type-specific knockout mice, we could show that endothelial CD73 is involved in glucose uptake and endogenous lipid synthesis in WAT but is dispensable for adaptive thermogenesis. Mechanistic cell culture studies indicates that intracellular conversion of adenosine to AMP by adenosine kinase inhibits fatty acid synthesis by the inactivation of the DNL transcription factor ChREBP. These finding provide evidence that vascular endothelial cells of adipose tissues are equipped to form extracellular adenosine as potential signalling molecule modulating adipocyte glucose and lipid metabolism (summarized in a model shown in **Figure 4F**).

In WAT and BAT, energy uptake and storage versus fatty acid release and heat production are determined by various signalling pathways, which are regulated primarily by insulin and norepinephrine (10, 25, 57). It is therefore of note that in the current study the specific deletion of endothelial CD73 affects nutrient uptake and lipid metabolism in adipose tissues. While endothelial CD73 deficiency had only minor effects on thermogenic adaptation to mild and severe cold conditions, it increases glucose uptake and subsequent DNL genes in WAT under thermoneutral conditions. The data suggest that extracellular adenosine generated by endothelial CD73 fine tunes energy acquisition pathways primarily under conditions of a low sympathetic tone. Importantly, DNL in WAT is associated with improved insulin sensitivity and therefore counteracts obesity-induced insulin resistance (31, 58, 59). Moreover, thermoneutral housing of mice that closely resembles human standard living conditions is described to decrease DNL in adipose tissues (60, 61), thereby potentially lowering the beneficial effect of endogenous lipid synthesis. Accordingly, our data suggest that inhibition of endothelial CD73 might prevent chronic inflammatory metabolic diseases triggered by excess fat deposition in WAT. Based on previous studies where adenosine supplementation and pharmacological activation of the adenosine receptor A2A and A2B activates thermogenic responses in WAT and BAT (41, 42), on the first view it might be surprising that endothelial CD73 did not have a profound impact on systemic energy expenditure and thermogenic adaptations of adipose tissues. This is, however, consistent with the observation that whole-body CD73-deficient mice have lower adenosine concentrations in BAT under basal conditions, but display increased local adenosine levels following electric field stimulation (42). Together these data suggest that CD73 activity modulates basal adenosine levels in adipose tissue but other sources might be more important for increasing extracellular adenosine levels under conditions of thermogenic stress. For instance, the glycosyl-phosphatidylinositol-anchored ectoenzyme tissue-nonspecific alkaline phosphatase (TNAP) can also generate extracellular adenosine from ATP and has been recently described to regulate thermogenesis in BAT (62). In addition, the abundance of extracellular adenine nucleotides could be regulated by export and import processes via specific transporters such as the equilibrative nucleoside transporter 1 (ENT1). In line, the adipose-specific ablation of ENT1 results in enhanced BAT activity and counteracts diet-induced obesity (43). Overall, extracellular adenosine acts as a paracrine factor regulating multiple functions within adipose tissues. However, the contribution of adenosine sources and the subsequent signalling pathways may vary depending on environmental conditions.

AMPK acts as critical energy sensor and homeostatic regulator of glucose and lipid metabolism in adipose depots (63–65). Enzyme activation depends on intracellular AMP levels, which in part is regulated by the phosphorylation of adenosine by ADK. Previous studies performed in primary hepatocytes and the intestinal epithelial cell line IEC-6 suggest that CD73-derived extracellular adenosine enters the cell and is rapidly converted to AMP via ADK. The resulting increase in intracellular AMP levels leads to AMPK activation (50, 51). This is consistent with the data of the current study showing that extracellular adenosine induces AMPK phosphorylation and its target ACC in white adipocytes, an effect that can be blunted by ADK inhibition. AMPK is known to phosphorylate ChREBP-alpha, which results in nuclear export of this transcription factor thereby preventing expression of its target genes such as *Chrebp-beta*, *Fasn* and *Acaca* (32, 53, 66). Here, we demonstrate that adenosine-induced AMPK phosphorylation was linked to reduced expression of DNL-regulating factors in primary white adipocytes. Correspondingly, endothelial CD73 deficiency, which most likely leads to lower extracellular adenosine levels, was associated with higher expression of *Chrebp* and DNL genes in WAT. In addition to DNL genes, ChREBP regulate systemic glucose metabolism by inducing the expression of genes that regulate glucose uptake and glycolysis (32, 66). This explains the higher glucose disposal rates into WAT of mice lacking CD73 in endothelial cells, as observed in the current study. In this way, ChREBP orchestrates glucose and lipid metabolism by providing glucose-derived acetyl-CoA as DNL precursor substrates. Overall, this study describes an intercellular adenosine-dependent signalling pathway in WAT that enables microvascular endothelial cells to coordinate glucose uptake and lipid metabolism in white adipocytes.

## Data availability statement

The original contributions presented in the study are included in the article/**Supplementary Material**. Further inquiries can be directed to the corresponding author.

## Ethics statement

The studies involving humans were approved by Ethics Committee of the Hamburg Chamber of Physicians. The studies were conducted in accordance with the local legislation and institutional requirements. The participants provided their written informed consent to participate in this study. The animal study was approved by Animal Welfare Officers at University Medical Center Hamburg-Eppendorf and Behörde für Gesundheit und Verbraucherschutz Hamburg. The study was conducted in accordance with the local legislation and institutional requirements.

## Author contributions

MJ: Conceptualization, Investigation, Methodology, Visualization, Writing – original draft, Writing – review & editing. IS: Investigation, Methodology, Writing – review & editing. MZ: Investigation, Methodology, Writing – review & editing. MH: Investigation, Methodology, Writing – review & editing. OM: Methodology, Resources, Writing – review & editing. AP: Funding acquisition, Methodology, Writing – review & editing. JH: Conceptualization, Funding acquisition, Investigation, Supervision, Validation, Writing – original draft, Writing – review & editing.
