# Supplementary material for: CD73-dependent generation of extracellular adenosine by vascular endothelial cells modulates de novo lipogenesis in adipose tissue
Source: Front Immunol. 2024 Jan 9;14:1308456. doi: 10.3389/fimmu.2023.1308456 (PMC10803534; doi:10.3389/fimmu.2023.1308456)
Supplement: Supplementary file 1 [file DataSheet_1.pdf]

Supplementary Material

CD73-dependent generation of extracellular adenosine by vascular endothelial cells modulates *de novo* lipogenesis in adipose tissue

Michelle Y. Jaeckstein[1], Isabell Schulze[1], Michael Wolfgang Zajac[1], Markus Heine[1], Oliver Mann[2], Alexander Pfeifer[3], Joerg Heeren[1]

[1]Department of Biochemistry and Molecular Cell Biology, University Medical Center Hamburg-Eppendorf, Hamburg, Germany

[2]Department of General, Visceral and Thoracic Surgery, University Medical Center Hamburg-Eppendorf, Hamburg, Germany

[3]Institute of Pharmacology and Toxicology, University Hospital, University of Bonn, Bonn, Germany

*** Correspondence:** Joerg Heeren: heeren@uke.de

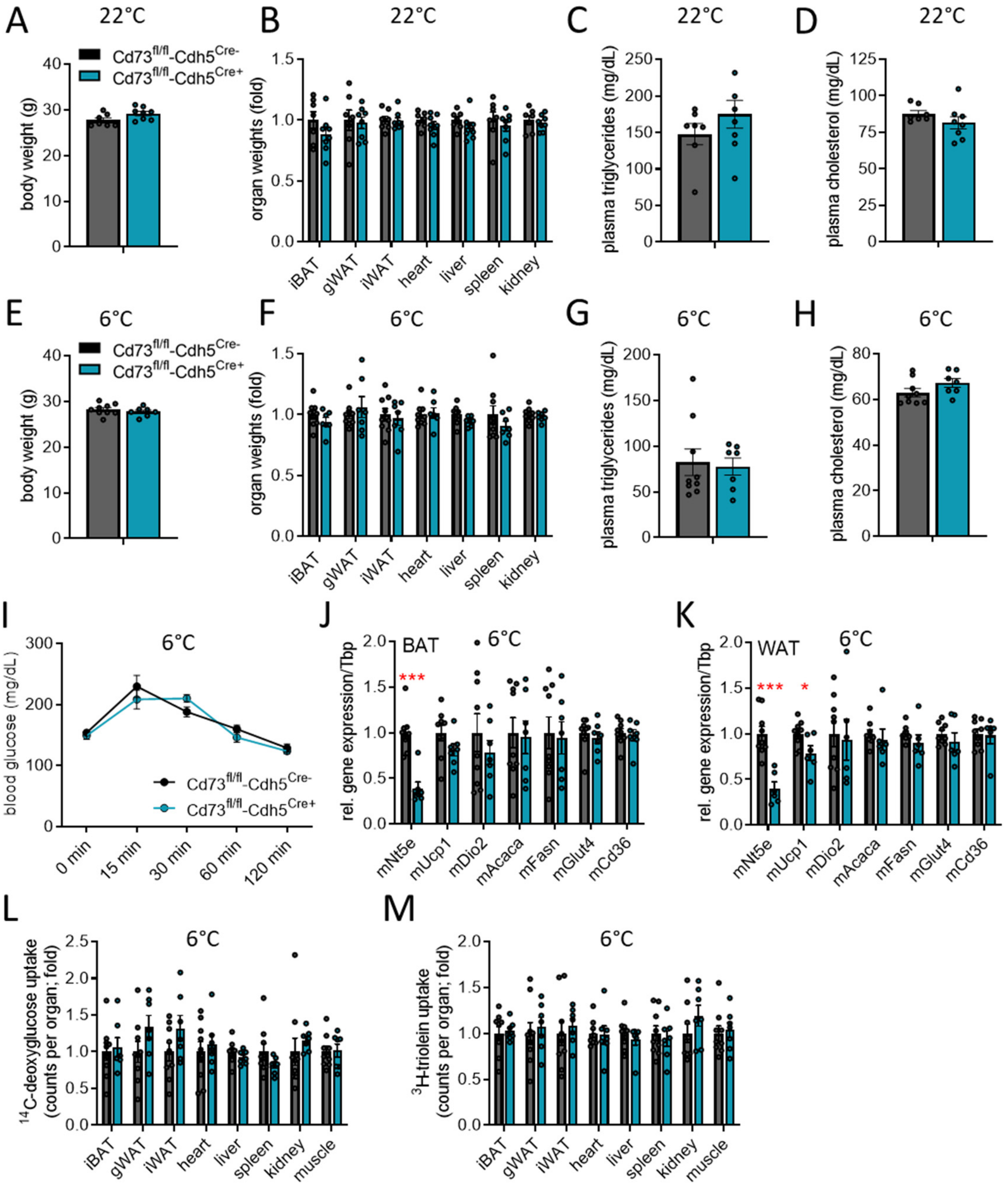

Supplementary Figure 1. Endothelial cell-specific CD73 deletion has minor effects on energy metabolism in cold-stressed mice (related to Figure 2). **(A-D)** Male endothelial cell-specific CD73 knockout mice ($CD73^{fl/fl}$-$Cdh5^{Cre+}$) and controls ($CD73^{fl/fl}$-$Cdh5^{Cre-}$) were housed at 22°C or **(E-M)** at 6°C for 1 week. **(A)** Body weights, **(B)** organ weights, **(C)** plasma triglycerides and **(D)** plasma cholesterol levels (n=7-8). **(E)** Body weights, **(F)** organ weights, **(G)** plasma triglycerides and **(H)** plasma cholesterol levels (n=7-9). **(I)** Blood glucose levels during oral glucose fat tolerance test (OGFT) (n=7-9). **(J)** BAT (n=7-9) and **(K)** WAT gene expression (n=6-9). **(L)** Uptake of ^{14}C-deoxyglucose (DOG) and **(M)** ^{3}H-triolein per total organ (n=7-9). Data are presented as mean values ± SEM. *p < 0.05, **p < 0.01, ***p < 0.001 by Student's t test. Overall, no major differences in various metabolic parameters were detected between genotypes when mice were housed at mild cold (22°C) or sustained exposure at 6°C.

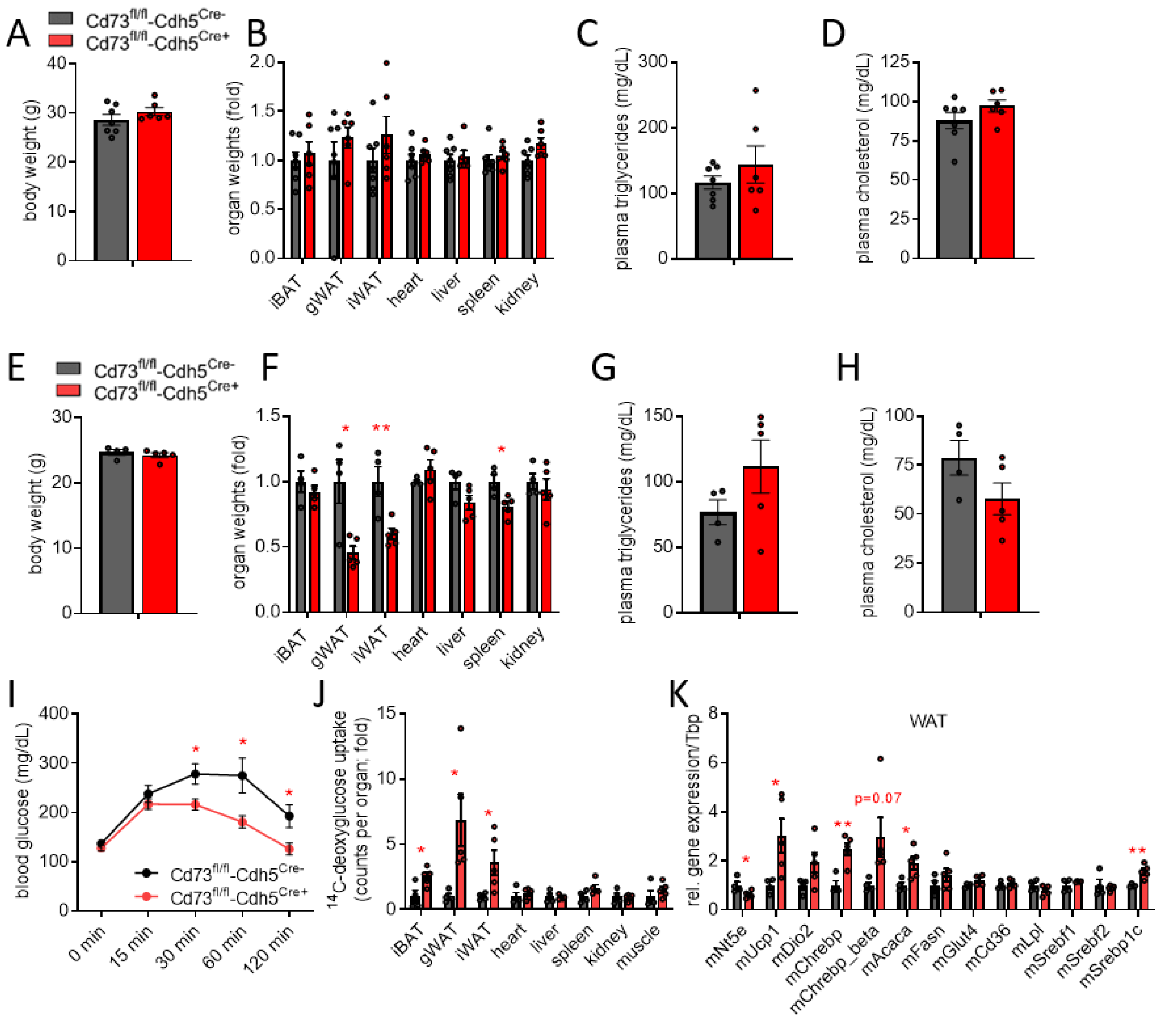

Supplementary Figure 2. In thermoneutrality, lack in endothelial CD73 results in higher glucose uptake and DNL genes in WAT (related to Figure 3). **(A-D)** Male endothelial cell-specific CD73 knockout mice (CD73$^{fl/fl}$-Cdh5^{Cre+}) and controls (CD73$^{fl/fl}$-Cdh5^{Cre-}) were housed at thermoneutrality (30°C). **(A)** Body weights, **(B)** organ weights, **(C)** plasma triglycerides and **(D)** plasma cholesterol levels were similar comparing CD73$^{fl/fl}$-Cdh5^{Cre+} and CD73$^{fl/fl}$-Cdh5^{Cre-} mice acclimated to 30°C (n=6-7). **(E-K)** Female endothelial cell-specific CD73 knockout mice (CD73$^{fl/fl}$-Cdh5^{Cre+}) and controls (CD73$^{fl/fl}$-Cdh5^{Cre-}) were housed at thermoneutrality (30°C). **(E)** Body weights, **(F)** organ weights, **(G)** plasma triglycerides and **(H)** plasma cholesterol levels of the CD73$^{fl/fl}$-Cdh5^{Cre+} and CD73$^{fl/fl}$-Cdh5^{Cre-} acclimated to 30°C (n=4-5). Female CD73$^{fl/fl}$-Cdh5^{Cre+} mice displayed improved **(I)** blood glucose levels during oral glucose fat tolerance test (OGFT) (n=4-5), higher (**J**) uptake of ^{14}C-deoxyglucose (DOG) per total organ (n=4-5) as well as higher (**K**) WAT gene expression of *de novo* lipogenesis (DNL) genes (n=4-5). Data are presented as mean values ± SEM. *p < 0.05, **p < 0.01, ***p < 0.001 by Student's t test. Overall, female and male mice lacking endothelial CD73 displayed a similar metabolic phenotype when housed at thermoneutrality.

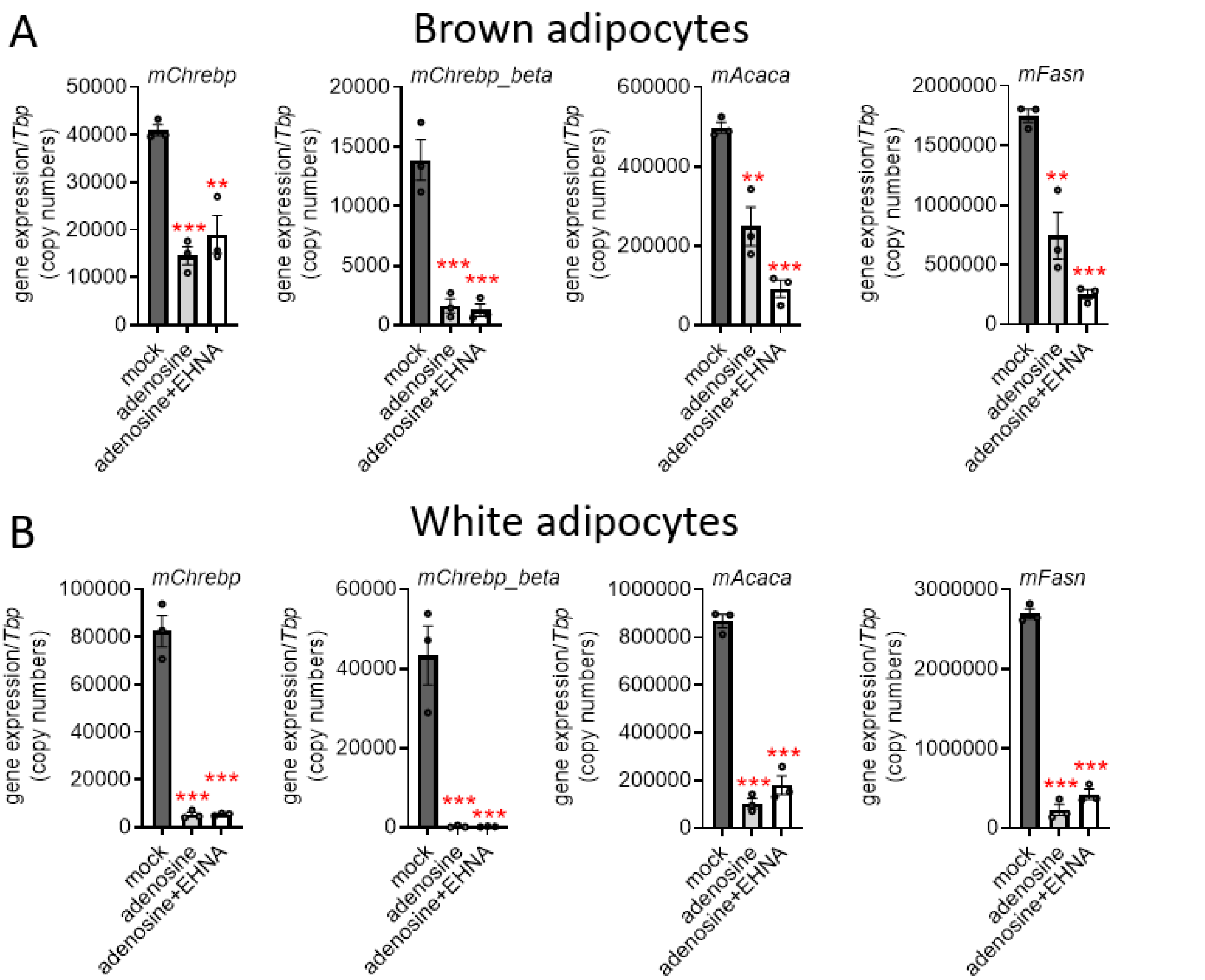

Supplementary Figure 3. In thermoneutrality, lack in endothelial CD73 results in higher glucose uptake and DNL genes in WAT (related to Figure 3). (**A-B**) Primary stromal-vascular cell (SVC)-derived brown and white adipocytes were differentiated in the absence (mock) or presence of adenosine and the adenosine deaminase (ADA) inhibitor EHNA. In both conditions, adenosine supplementation lowers gene expression of *de novo* lipogenesis (DNL) genes in differentiated primary (**A**) brown and (**B**) white adipocytes (n=3). Data are presented as mean values ± SEM. *p < 0.05, **p < 0.01, ***p < 0.001 by ANOVA comparing mock versus indicated treatments.

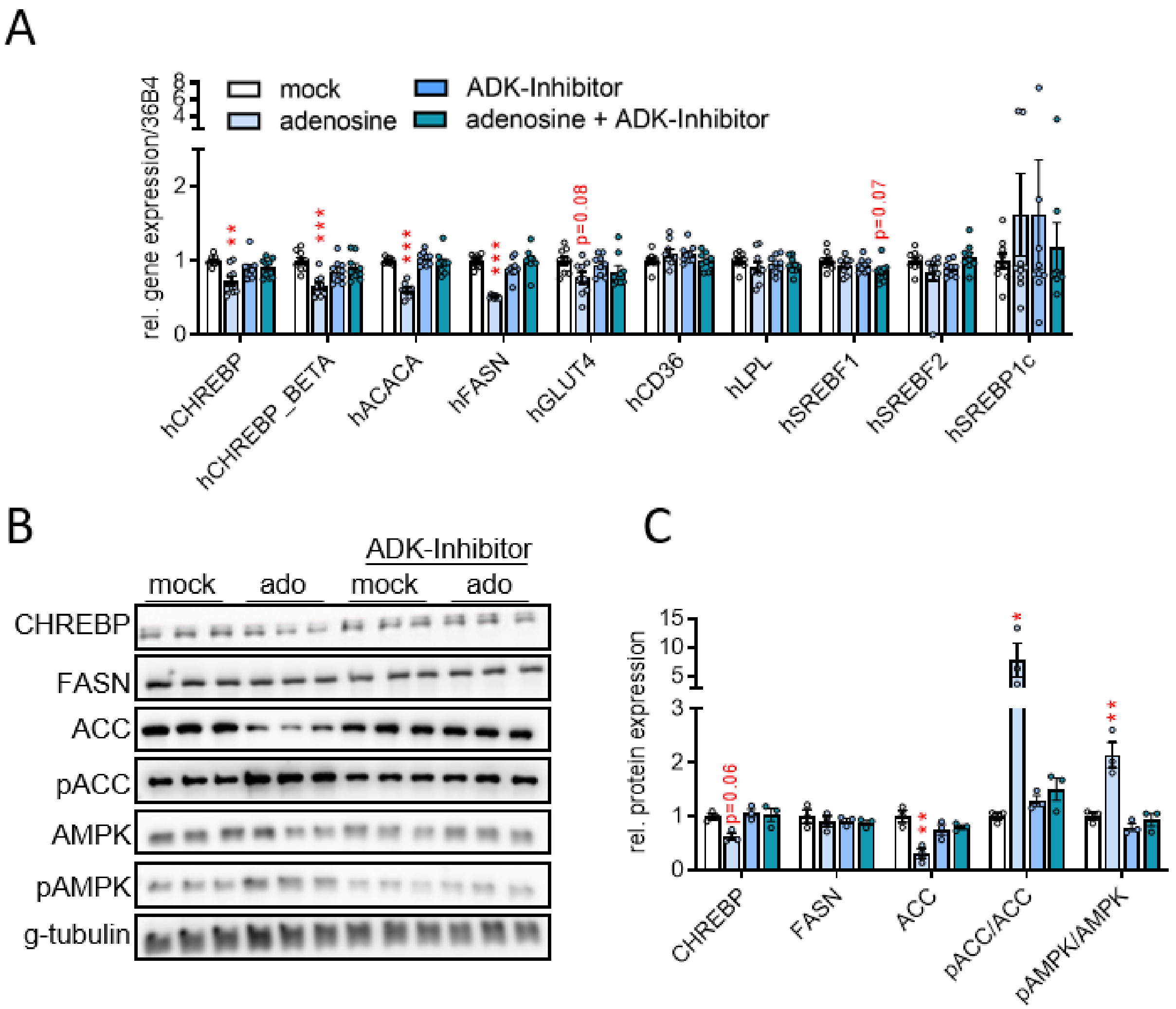

Figure S4 Extracellular adenosine lowers *de novo* lipogenesis (DNL) marker expression via an adenosine kinase (ADK)-dependent axis (related to Figure 4). **(A-C)** Human primary stromal-vascular cell (SVC)-derived white adipocytes were differentiated in the absence (mock) or presence of adenosine. ADK was inhibited by ABT-702. **(A)** Effect of adenosine and ADK inhibitor on gene expression in human primary white adipocytes (n=9). **(B)** Effect of adenosine and ADK inhibitor on protein expression in human primary white adipocytes. **(C)** Quantification of proteins shown in **(B)** (n=3). Data are presented as mean values ± SEM. *p < 0.05, **p < 0.01, ***p < 0.001 by ANOVA comparing mock versus indicated treatments. These data show that the lowering effect of adenosine on DNL gene and protein expression in human adipocytes can be rescued by ADK inhibition.

Supplementary Table 1

**Figure 1**				*p*	*n*
	**A**	*Ucp1*	CD11b+ vs. flow through	0.0026	4
			CD31+ vs. flow through	0.0070	4
		Emr1	CD11b+ vs. CD31+	<0.0001	4
			CD11b+ vs. flow through	<0.0001	4
		Gpihbp1	CD11b+ vs. CD31+	<0.0001	4
			CD31+ vs. flow through	<0.0001	4
		Nt5e	CD11b+ vs. CD31+	<0.0001	4
			CD31+ vs. flow through	<0.0001	4
	**B**	*Adipoq*	CD11b+ vs. flow through	0.0040	4
			CD31+ vs. flow through	0.0040	4
		Emr1	CD11b+ vs. CD31+	<0.0001	4
			CD11b+ vs. flow through	<0.0001	4
		Gpihbp1	CD11b+ vs. CD31+	<0.0001	4
			CD31+ vs. flow through	<0.0001	4
		Nt5e	CD11b+ vs. CD31+	<0.0001	4
			CD11b+ vs. flow through	<0.0001	4
			CD31+ vs. flow through	<0.0001	4
**Figure 2**					
	**A**	*Nt5e*	BAT	1.23266E-06	7-8
		Nt5e	WAT	0.004835242	7-8
	**B**	*Nt5e*	CD31+	0.074916216	3
		Nt5e	flow through	0.173795046	3
	**C**	*Nt5e*	CD31+	4.85312E-05	3
		Nt5e	flow through	0.134805387	3
	**E**	^{14}C-DOG counts	iBAT	0.01160872	7-8
			heart	0.01309832	7-8
			liver	0.06384755	7-8
			muscle	0.04410429	7-8
	**F**	^{3}H-triolein counts	iBAT	0.02679433	7-8
			heart	0.00103237	7-8
			liver	0.07872564	7-8
			muscle	0.02468439	7-8
**Figure 3**					
	**B**	^{14}C-DOG counts	gWAT	0.01432149	5-7
			iWAT	0.06956811	5-7
	**D**	*Nt5e*	BAT	7.12114E-08	6-7
		Acaca	BAT	0.064915836	6-7
		Fasn	BAT	0.054143605	6-7
	**E**	*Nt5e*	WAT	0.000319205	6-7
		Chrebp	WAT	0.038039198	6-7
		Chrebp_beta	WAT	0.001981467	6-7
		Acaca	WAT	2.37383E-05	6-7
		Fasn	WAT	0.000313221	6-7
		Lpl	WAT	0.01961520	6-7
		Srebf1	WAT	0.011069515	6-7
		Srebp1c	WAT	0.017503163	6-7
	**F**	*Chrebp*	brown adipocytes	0.000311719	3

		Chrebp_beta	brown adipocytes	0.002460273	3
		Acaca	brown adipocytes	0.007923024	3
		Fasn	brown adipocytes	0.007755689	3
		Glut4	brown adipocytes	9.78934E-05	3
		Cd36	brown adipocytes	0.023776031	3
		Srebp1c	brown adipocytes	0.075925701	3
	**G**	*Chrebp*	white adipocytes	0.000332416	3
		Chrebp_beta	white adipocytes	0.004424263	3
		Acaca	white adipocytes	2.53471E-05	3
		Fasn	white adipocytes	1.30705E-05	3
		Glut4	white adipocytes	3.18115E-05	3
		Cd36	white adipocytes	0.000275414	3
		Lpl	white adipocytes	0.000275582	3
		Srebp1c	white adipocytes	3.97266E-05	3
**Figure 4**					
	**A**	(*p*-values shown for comparing mock versus indicated treatments)			
		Chrebp	mock vs. adenosine	<0.0001	9
		Chrebp_beta	mock vs. adenosine	<0.0001	9
		Acaca	mock vs. adenosine	<0.0001	9
		Fasn	mock vs. adenosine	<0.0001	9
		Fasn	mock vs. adenosine + ADK-Inhibitor	0.0783	9
		Glut4	mock vs. adenosine	<0.0001	9
		Cd36	mock vs. adenosine	<0.0001	9
		Lpl	mock vs. adenosine	<0.0001	9
		Srebp1c	mock vs. adenosine	<0.0001	9
	**C**	(*p*-values shown for comparing mock versus indicated treatments)			
		CHREBP	mock vs. adenosine	<0.0001	9
		FASN	mock vs. adenosine	<0.0001	9
		FASN	mock vs. ADK-Inhibitor	0.0360	9
		ACC	mock vs. adenosine	0.0002	9
		pACC/ACC	mock vs. adenosine	0.0014	9
		pAMPK/AMPK	mock vs. adenosine	<0.0001	9
	**E**	FASN	WAT	0.01524376	6-7
		ACC	WAT	8.9697E-05	6-7
		pAMPK/AMPK	WAT	0.03523164	6-7
**Figure S1**					
	**L**	*Nt5e*	BAT	3.04509E-05	7-9
	**M**	*Nt5e*	WAT	0.000179753	6-9
		Ucp1	WAT	0.029695542	6-9
**Figure S2**					
	**F**	organ weight	gWAT	0,010567212	4-5
		organ weight	iWAT	0,008896782	4-5
		organ weight	spleen	0,015714105	4-5
	**I**	blood glucose	30 min	0,025982881	4-5
		blood glucose	60 min	0,028267219	4-5
		blood glucose	120 min	0,031275499	4-5
	**J**	^{14}C-DOG counts	iBAT	0,025519385	4-5
		^{14}C-DOG counts	gWAT	0,035051857	4-5
		^{14}C-DOG counts	iWAT	0,049007696	4-5
	**K**	*Nt5e*	WAT	0,04578299	4-5

		Ucp1	WAT	0,036957031	4-5
		Chrebp	WAT	0,002873442	4-5
		Chrebp_beta	WAT	0,071065193	4-5
		Acaca	WAT	0,024952153	4-5
		Srebp1c	WAT	0,004854643	4-5
**Figure S3**					
	**E**	(*p*-values shown for comparing mock versus indicated treatments)			
		Chrebp	mock vs. adenosine	0.0010	3
		Chrebp	mock vs. adenosine + EHNA	0.0027	3
		Chrebp_beta	mock vs. adenosine	0.0005	3
		Chrebp_beta	mock vs. adenosine + EHNA	0.0004	3
		Acaca	mock vs. adenosine	0.0035	3
		Acaca	mock vs. adenosine + EHNA	0.0002	3
		Fasn	mock vs. adenosine	0.0024	3
		Fasn	mock vs. adenosine + EHNA	0.0003	3
	**F**	(*p*-values shown for comparing mock versus indicated treatments)			
		Chrebp	mock vs. adenosine	<0.0001	3
		Chrebp	mock vs. adenosine + EHNA	<0.0001	3
		Chrebp_beta	mock vs. adenosine	0.0010	3
		Chrebp_beta	mock vs. adenosine + EHNA	0.0010	3
		Acaca	mock vs. adenosine	<0.0001	3
		Acaca	mock vs. adenosine + EHNA	<0.0001	3
		Fasn	mock vs. adenosine	<0.0001	3
		Fasn	mock vs. adenosine + EHNA	<0.0001	3
**Figure S4**					
	**A**	(*p*-values shown for comparing mock versus indicated treatments)			
		CHREBP	mock vs. adenosine	0.0014	9
		CHREBP_BETA	mock vs. adenosine	0.0002	9
		ACACA	mock vs. adenosine	<0.0001	9
		FASN	mock vs. adenosine	<0.0001	9
		GLUT4	mock vs. adenosine	0.0761	9
		SREBF1	mock vs. adenosine + ADK-inhibitor	0.0704	9
	**C**	(*p*-values shown for comparing mock versus indicated treatments)			
		CHREBP	mock vs. adenosine	0.0563	3
		ACC	mock vs. adenosine	0.0029	3
		pACC/ACC	mock vs. adenosine	0.0426	3
		pAMPK/AMPK	mock vs. adenosine	0.0020	3
